# Sciatic Nerve Neuropathy Secondary to Sacral Pressure Ulcer Following COVID-19: A Case Report

**DOI:** 10.7759/cureus.84670

**Published:** 2025-05-23

**Authors:** Jorge F Tuma, Pedro Henrique Marte de Arruda Sampaio, Paulo Tuma Jr.

**Affiliations:** 1 General Surgery, Hospital Israelita Albert Einstein, São Paulo, BRA; 2 Neurology, Hospital das Clínicas, Faculdade de Medicina, Universidade de São Paulo (HC-FMUSP), São Paulo, BRA; 3 Plastic Surgery, Hospital das Clínicas, Faculdade de Medicina, Universidade de São Paulo (HC-FMUSP), São Paulo, BRA

**Keywords:** case report, covid-19, gluteus maximus flap, icu complications, negative pressure therapy, neuropathic pain, pressure ulcer, reconstructive surgery, sciatic neuropathy, v-y flap

## Abstract

We report the case of a previously healthy 25-year-old man who developed severe SARS-CoV-2 infection requiring prolonged mechanical ventilation. Following extubation, he presented with peripheral sensory deficits and a sacral pressure ulcer. He was later admitted to our service with fever and a Grade IV ulcer, which required surgical debridement, negative pressure wound therapy, and definitive reconstruction using a V-Y gluteal flap. Examination of the right leg at admission demonstrated a neuropathic pain pattern, with neurological evaluation and imaging confirming distal sensorimotor polyneuropathy and chronic proximal sciatic neuropathy. Treatment with pregabalin led to complete resolution of symptoms, with no neurological deficit or residual pain identified during follow-up. This report suggests a plausible intersection between pressure-related and neurological complications in critically ill COVID-19 patients and highlights the importance of early preventive strategies such as frequent repositioning and multidisciplinary ICU care.

## Introduction

During the early phases of the COVID-19 pandemic, a range of neurological complications, including Guillain-Barré syndrome and critical illness myopathy, were frequently observed in severely ill patients, as reported by Mao et al. in 36.4% of hospitalized cases in Wuhan [1,2]. Furthermore, prolonged immobility during intensive care is a well-recognized risk factor for both pressure ulcer (PU) development and peripheral neuropathy, further compounding morbidity [3-5]. The coexistence of these complications poses significant challenges to recovery. This report describes a rare clinical scenario involving chronic sciatic neuropathy in association with a sacral PU in a patient with severe COVID-19 infection, emphasizing the need for early preventive strategies and comprehensive care in intensive care settings.

## Case presentation

A previously healthy 25-year-old man was admitted to another hospital on March 31, 2020, with SARS-CoV-2 infection that rapidly progressed to respiratory failure and systemic shock. COVID-19 was confirmed by RT-PCR of a nasal swab. He required intensive care unit (ICU) admission and orotracheal intubation, remaining critically ill on mechanical ventilation for 15 days. During this period, he underwent prone positioning and received high-dose vasopressor support.

Following extubation, he reported bilateral distal hypoesthesia and paresthesia in the lower limbs, in addition to a 27 kg weight loss. Thirteen days later, he was discharged with a sacral PU, which had been managed conservatively with papain-based chemical debridement and ciprofloxacin. Initially, his sensory symptoms improved, persisting only in the left toes. However, he began to experience nocturnal dysesthesia and painful shocks in the right foot, along with worsening of the sacral ulcer.

**Figure 1:**
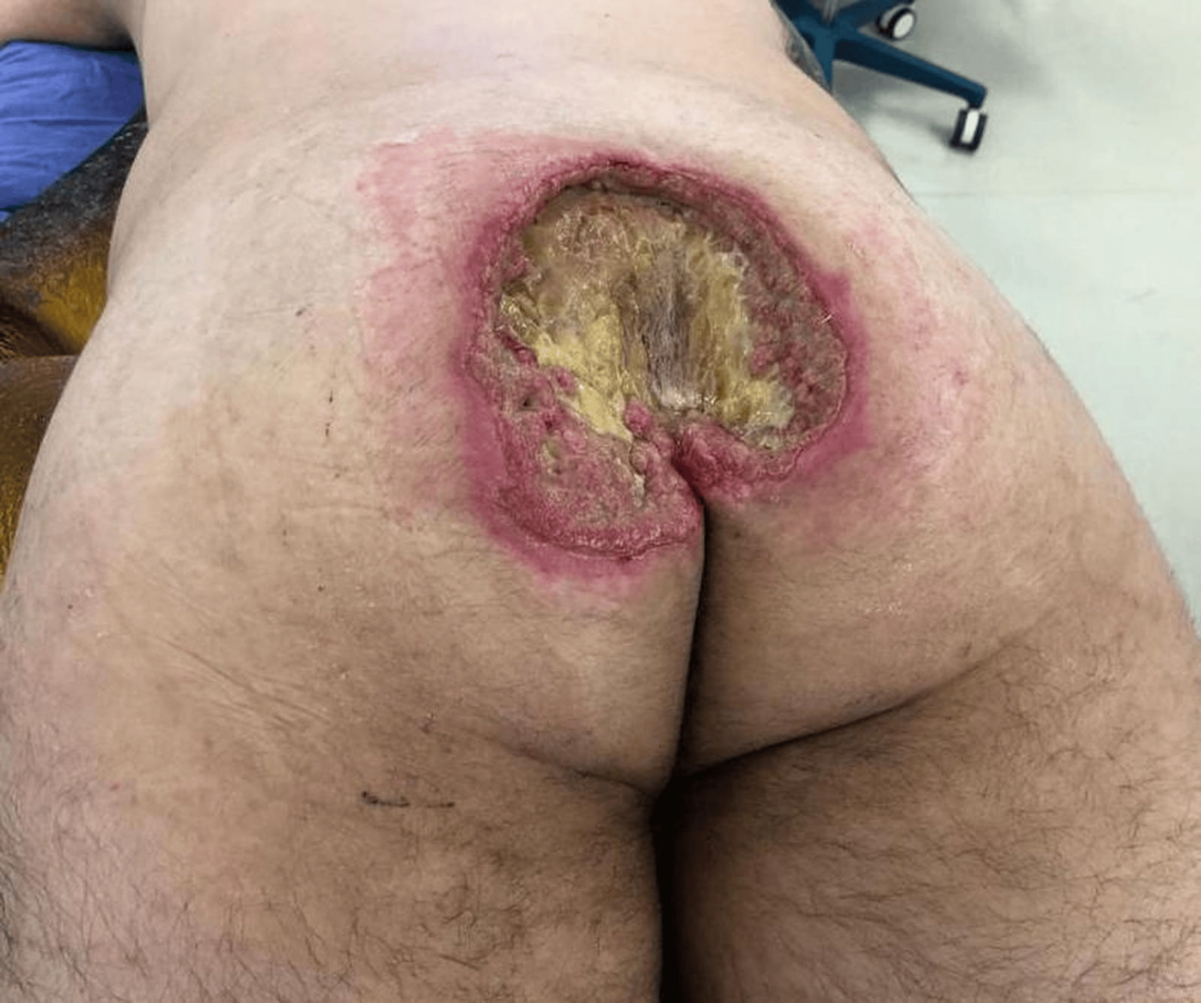
Sacral pressure ulcer on admission

**Figure 2:**
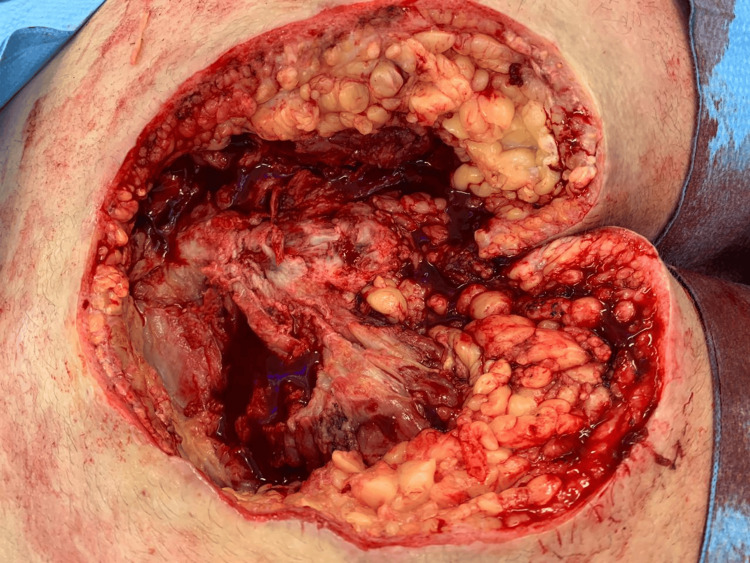
Intraoperative view post-debridement

**Figure 3:**
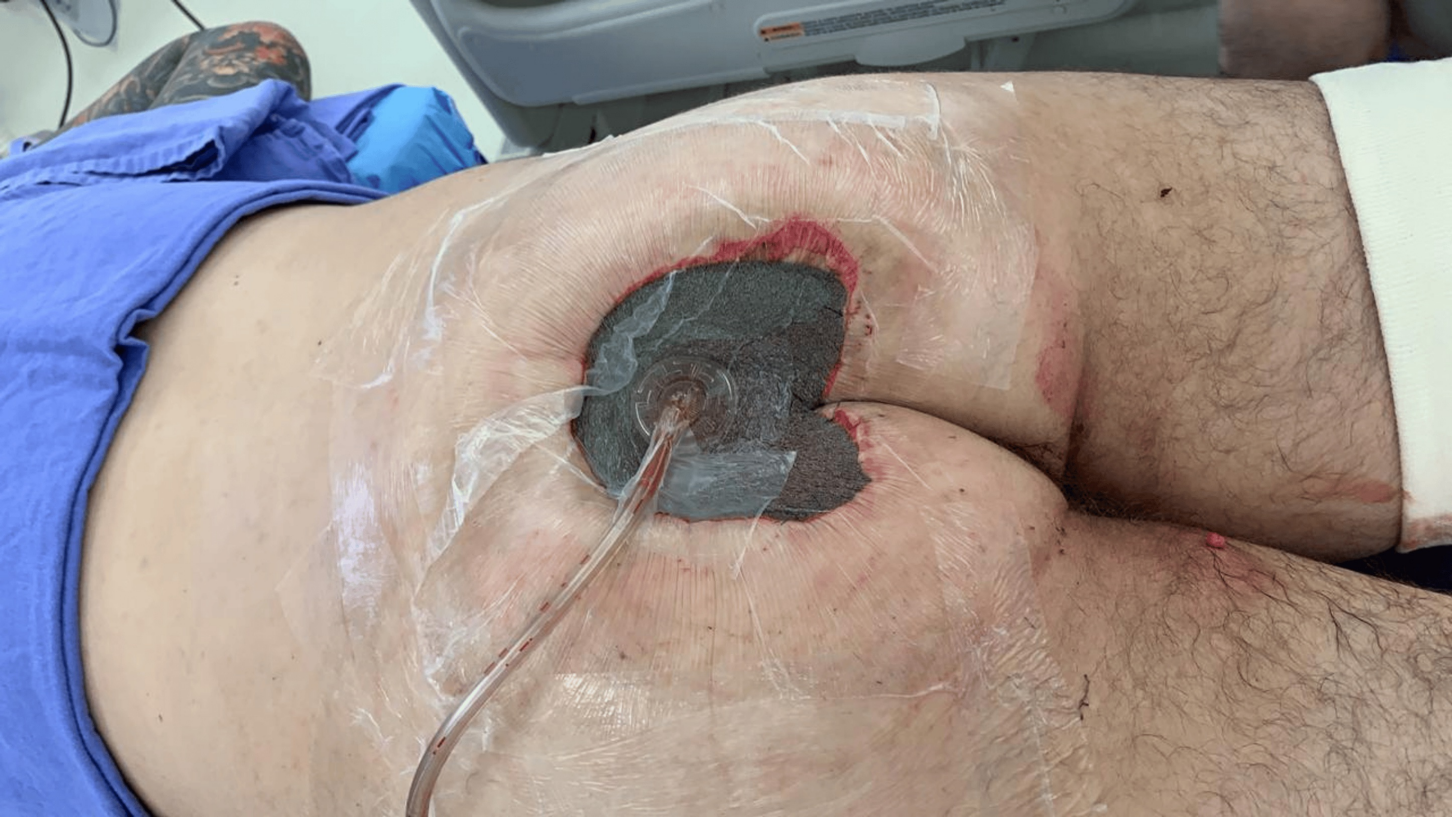
Negative pressure therapy applied

**Figure 4:**
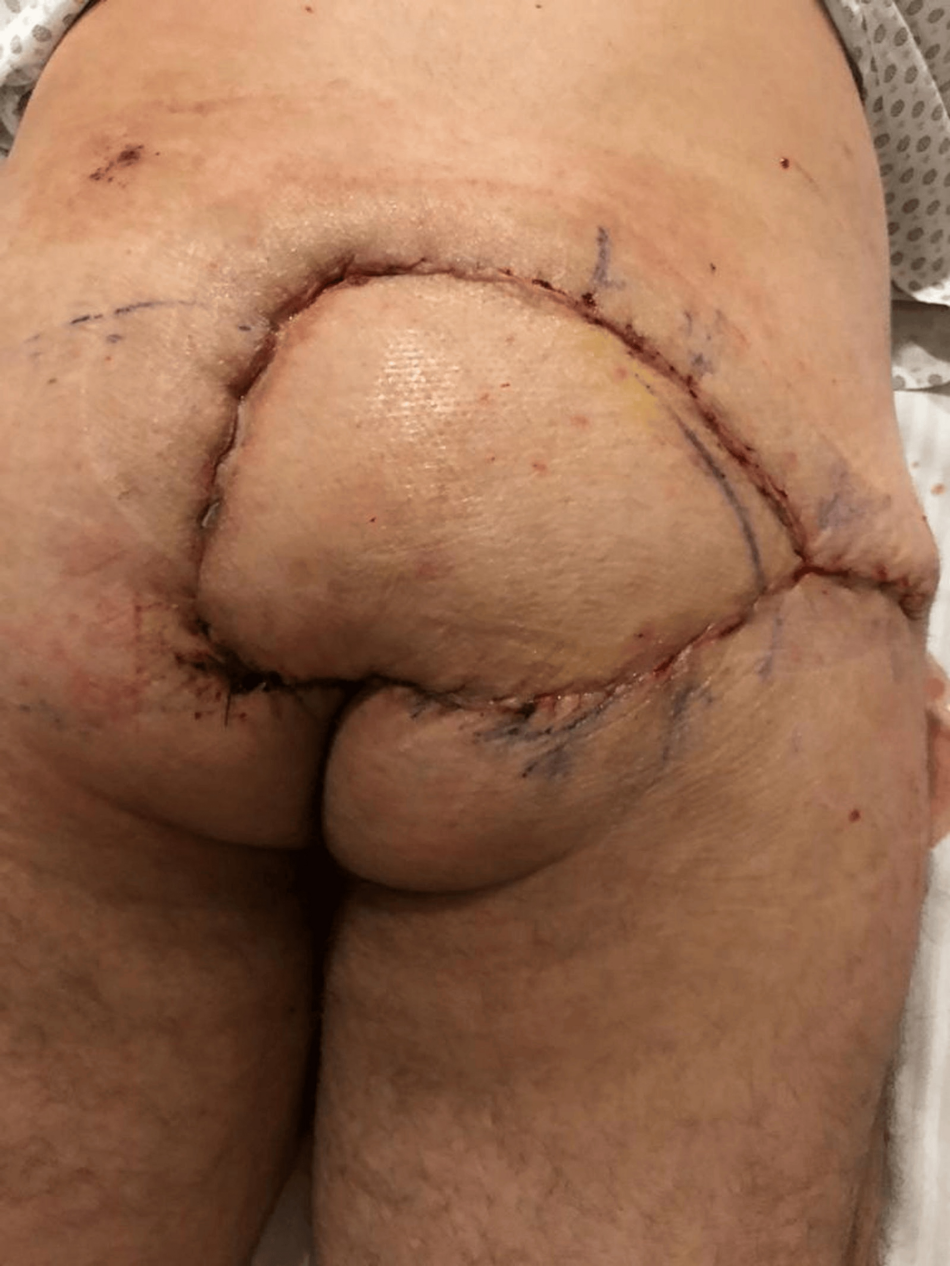
V-Y gluteal flap

On May 25, 2020, he was admitted to a tertiary care center with fever and malaise. Physical examination revealed a Grade IV sacral ulcer measuring approximately 20 cm in diameter (Figure 1). Blood tests demonstrated signs of systemic infection, including leukocytosis and elevated inflammatory markers (Table 1). He received intravenous daptomycin and ceftazidime/avibactam and underwent surgical debridement two days later, with extensive removal of necrotic and fibrotic tissue (Figure 2). Negative pressure wound therapy was initiated (Figure 3), and culture results revealed meropenem-resistant Pseudomonas aeruginosa, Enterococcus faecalis, Parabacteroides distasonis, and Prevotella timonensis. Based on sensitivity, antimicrobial therapy was switched to daptomycin and polymyxin, later replaced by ceftolozane/tazobactam due to neurotoxicity. Five days after debridement, definitive closure was achieved with a V-Y right gluteal flap (Figure 4), with satisfactory postoperative evolution, appropriate tissue integration, and no recorded complications.

**Table 1:** Laboratory findings at hospital admission WBC: white blood cells; CRP: C-reactive protein; Hb: hemoglobin; Ht: hematocrit; INR: international normalized ratio; aPTT: activated partial thromboplastin time; AST: aspartate aminotransferase; ALT: alanine aminotransferase.

Parameter	Patient Value	Reference Range	Unit
Hemoglobin (Hb)	13.4	13.0 – 17.0	g/dL
Hematocrit (Ht)	39.7	40 – 50	%
White blood cells (WBC)	15,000	4,000 – 11,000	cells/mm³
Platelets (Plt)	297,000	150,000 – 400,000	cells/mm³
C-reactive protein (CRP)	30	< 5	mg/L
Potassium (K⁺)	3.6	3.5 – 5.0	mEq/L
Sodium (Na⁺)	137	135 – 145	mEq/L
Ionized calcium (Ca²⁺)	1.2	1.1 – 1.3	mmol/L
Magnesium (Mg²⁺)	1.8	1.6 – 2.4	mg/dL
Creatinine (Cr)	0.8	0.6 – 1.3	mg/dL
Urea (BUN)	33	10 – 50	mg/dL
INR	1.1	0.8 – 1.2	—
aPTT (TTPA)	37.5	25 – 35	seconds
AST (TGO)	23	10 – 40	U/L
ALT (TGP)	55	7 – 56	U/L
Total bilirubin	0.44	0.2 – 1.2	mg/dL
Direct bilirubin	0.21	< 0.3	mg/dL
Indirect bilirubin	0.23	< 1.0	mg/dL

**Figure 5:**
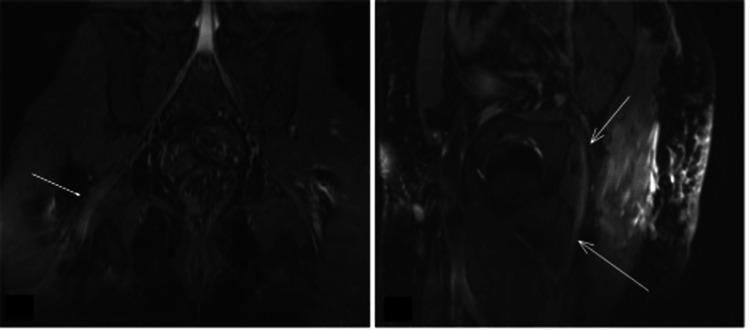
MRI sciatic neurography showing T2 hyperintensity in the right sciatic nerve

Due to persistent right leg neuropathic pain, a neurology consultation was obtained. Motor function, deep tendon reflexes, plantar responses, and vibration sense were preserved. However, tactile and pinprick sensations were reduced in the left toes, and dysesthesia was noted over the dorsal aspect of the right foot. Electromyography revealed a right-sided distal sensorimotor axonal polyneuropathy with superimposed chronic proximal sciatic neuropathy. The findings included reduced amplitudes and prolonged distal latencies in muscles innervated by the sciatic nerve. MRI neurography corroborated the electrophysiological findings, demonstrating T2 hyperintensity in the right sciatic nerve just beyond the greater sciatic foramen, with no evidence of nerve enlargement, fascicular swelling, or loss of fascicular pattern (Figure 5). Pregabalin 75 mg at bedtime was initiated, resulting in complete resolution of symptoms within two weeks, with no neurological deficits or residual pain documented on follow-up. The patient completed a 14-day antibiotic course, remained afebrile, and inflammatory laboratory markers such as leukocyte count and C-reactive protein showed progressive improvement (Table 2), consistent with systemic recovery. His last clinical evaluation, performed six months after discharge, showed stable condition and no recurrence of symptoms.

**Table 2:** Laboratory findings at follow-up WBC: white blood cells; CRP: C-reactive protein; Hb: hemoglobin; Ht: hematocrit.

Parameter	Patient Value	Reference Range	Unit
Hemoglobin (Hb)	13.2	13.0 – 17.0	g/dL
Hematocrit (Ht)	40.1	40 – 50	%
White blood cells (WBC)	8000.0	4,000 – 11,000	cells/mm³
C-reactive protein (CRP)	8.0	< 5	mg/L
Potassium (K⁺)	3.8	3.5 – 5.0	mEq/L
Sodium (Na⁺)	136.0	135 – 145	mEq/L
Creatinine (Cr)	0.7	0.6 – 1.3	mg/dL
Urea (BUN)	12.0	10 – 50	mg/dL

## Discussion

Neurological manifestations became widely recognized during the early phases of the COVID-19 pandemic, including Guillain-Barré syndrome and critical illness myopathy. Mao et al. reported neurological symptoms in 36.4% of hospitalized COVID-19 patients in Wuhan [1]. Neuroinvasion may occur via hematogenous spread or trans-neuronal routes [2,3]. Patients requiring prolonged mechanical ventilation face elevated risks of critical illness neuropathy and myopathy due to disuse atrophy and type II muscle fiber degeneration [4,5].

Critically ill and immobilized patients are also at heightened risk for PUs, with prevalence rates up to threefold higher than in non-COVID ICU patients [6,7]. PUs develop when sustained pressure exceeds local perfusion, particularly over bony prominences [8,9]. The pro-inflammatory cytokine surge in COVID-19, especially IL-6 and TNF-α, may further contribute to tissue damage and ulcer formation [7]. In this case, the patient exhibited several known risk factors for PUs: prolonged immobility, obesity, moisture, and wound colonization [8,9].

Clinical examination revealed a Grade IV PU, characterized by extensive necrosis and fibrotic debris (Figure 1). This clinical scenario illustrates key principles guiding the treatment of advanced PUs. Surgical management of Stage III/IV PUs includes thorough debridement and reconstruction due to the potential for masked deeper tissue injury, often described as resembling an iceberg (Figure 2) [9,10]. Negative pressure therapy was used to prepare the wound bed, improving perfusion, reducing edema, promoting granulation, and controlling infection (Figure 3) [9,11-13]. Definitive closure commonly requires fasciocutaneous or musculocutaneous flaps, as grafts lack sufficient durability and primary closure is typically contraindicated [9,10]. In this case, a V-Y gluteal flap was performed for definitive coverage of the sacral pressure ulcer (Figure 4), a well-established technique for this region [10], selected for its robust perfusion, proximity, and ability to preserve major muscle function.

Pain is a natural stimulus for repositioning, serving as a protective mechanism against ulcer development. In conscious individuals, this nociceptive feedback promotes spontaneous movement and pressure relief. In unconscious or sedated patients, this reflex is absent, further increasing PU risk [6]. In the present case, early and consistent mobilization might have mitigated ulcer severity and prevented progression to a deep lesion. For this reason, frequent repositioning by healthcare teams is essential. Education and awareness among ICU staff play a crucial role in PU prevention and directly impact patient outcomes and quality of life [14].

## Conclusions

Severe respiratory infections requiring intensive care, such as SARS-CoV-2, often lead to prolonged hospital stays due to the intense systemic inflammatory response they provoke. Consequently, long-term immobility becomes a significant risk factor for the development of myopathy or neuropathy, further increasing morbidity in these patients. This case illustrates a rare and clinically relevant association between a sacral PU and chronic sciatic neuropathy, emphasizing the importance of recognizing neurological complications in immobilized individuals. Electromyography, neuroimaging, and a multidisciplinary approach, including neurology, surgery, and rehabilitation, play a key role in diagnosis, treatment planning, and functional recovery. Preventive strategies for PUs remain essential, with frequent patient mobilization and reinforcement to healthcare professionals regarding the relevance of this issue and its direct impact on patient quality of life.
